# High-Resolution Tactile-Sensation Diagnostic Imaging System for Thyroid Cancer

**DOI:** 10.3390/s23073451

**Published:** 2023-03-25

**Authors:** So-Hyun Cho, Su-Min Lee, Na-Young Lee, Byoung Chul Ko, Hojeong Kim, Dae-Jin Jang, Jong-Ha Lee

**Affiliations:** 1 Department of Biomedical Engineering, School of Medicine, Keimyung University, Daegu 1095, Republic of Korea; 2 Department of Computer Engineering, Keimyung University, Daegu 1095, Republic of Korea; 3 Industry-Academic Cooperation Foundation, Keimyung University, Daegu 1095, Republic of Korea

**Keywords:** tactile sensor, non-invasive diagnosis, artificial intelligence, personal health record, elastic optical wave

## Abstract

In this study, we propose the direct diagnosis of thyroid cancer using a small probe. The probe can easily check the abnormalities of existing thyroid tissue without relying on experts, which reduces the cost of examining thyroid tissue and enables the initial self-examination of thyroid cancer with high accuracy. A multi-layer silicon-structured probe module is used to photograph light scattered by elastic changes in thyroid tissue under pressure to obtain a tactile image of the thyroid gland. In the thyroid tissue under pressure, light scatters to the outside depending on the presence of malignant and positive properties. A simple and easy-to-use tactile-sensation imaging system is developed by documenting the characteristics of the organization of tissues by using non-invasive technology for analyzing tactile images and judging the properties of abnormal tissues.

## 1. Introduction

The thyroid is an endocrine gland that surrounds the lower airway of the cartilaginous bone and protrudes forward from the middle of the neck. As the largest endocrine gland in the human body, it is not palpable under normal conditions, but tends to swell when abnormalities occur. The thyroid plays an essential role in the normal functioning of all the organs in the body.

According to the National Cancer Information Center [1], thyroid cancer ranked first among all the incidences of cancer in 2020, accounting for 21.5%. It is particularly common in women; however, as of 2020, it was the most common type of cancer among women, it was also the fourth most common among men—indicating a high incidence rate regardless of gender. Although there are several risk factors, including radiation exposure, hormone imbalance, and family history, the exact cause is still unknown. Although thyroid cancer has a low recurrence, since most patients survive for a long time, recurrence may still occur, and the possibility of metastasis to other organs also increases with time. Therefore, it is crucial to establish accurate methods for follow-up and early diagnosis. Currently, there is no proven method for preventing thyroid disease. However, since calcified tissues have a high propensity to transform into malignant cells, the early diagnosis of calcified tissues is a good proxy and is very beneficial for preventing thyroid cancer.

In this study, we investigate a low-cost portable device that can be utilized as a primary screening tool that measures tactile sensation using high-resolution tactile images. Usually, palpation is used as a diagnostic tool for thyroid cancer, but it is challenging to quantify human touch. For this device, a probe module was designed using a multilayer silicon structure that mimics human skin and utilizes reflected light. The probe module captures tactile images of the thyroid tissue by photographing dispersed light according to variations in the elasticity of thyroid tissues in response to pressure applied during scanning. As a result of this study, a simple and easy-to-use Tactile Sensation Imaging System (TSIS) for thyroid cancer was developed and validated through experiments. Using the TIR principle, light scatter was photographed to yield high-resolution tactile images inside a multilayer optical-wave guide mimicking human skin. Utilizing wave optics, the TIR was shown to be feasible and revealed clear tactile images. It allows the remote medical treatment of thyroid cancer, as well as frequent follow-up tests and recur-rent monitoring. Moreover, the non-invasive computerized self-examination device does not cause radiation exposure and can be used to simply examine the thyroid without constraints on time or location. The TSIS was developed for screening purposes to confirm the presence or progression and regression of tumors. Additional research is underway to diagnose whether tumors are malignant or benign using the TSIS.

### 1.1. Related Works

As the thyroid gland is not visible to most people and thyroid cancer has no distinct symptoms, it tends to be overlooked, even if a lump develops in the thyroid gland. Diagnosing the early stage of a tumor or nodule in the thyroid is also quite difficult. Thyroid cancer is primarily diagnosed by ultrasound imaging, biopsy, and various scanning techniques, such as computed tomography (CT), radioactive iodine scan, and positron emission tomography (PET). The current technologies used for diagnosing thyroid cancer have limitations that may be hazardous to the body under testing, such as radiation exposure. Regarding the thyroid gland, self-examination or palpation plays a primary role in detecting a tumor or lump. However, when using palpation, naturally, it is difficult to determine or digitally document the degree of malignancy. The palpation of the thyroid gland is highly subjective in nature and depends on the thyroid type and nodule depth.

Various methods for characterizing thyroid tissue have been proposed recently. Standard forms of diagnosis for evaluating malignant tumors in thyroid nodules include ultrasonography and fine-needle-aspiration cytology (FNAC)—serving to avoid unnecessary surgery for benign diseases or prevent malignant nodules’ disappearance. However, new methodologies are required to improve the diagnostic sensitivity and accuracy of FNAC. Thyroid imaging tests, including thyroid-isotope testing and thyroid scans, are used to determine malignancy by evaluating the presence of radioactive iodine in the thyroid nodule. In this method, the presence or absence of thyroid abnormalities is examined 18 h after the patient orally ingests a radioactive isotope of iodine (I-123) [2].

Magnetic resonance imaging (MRI), a non-invasive diagnostic tool, can create 2D or 3D images of the thyroid gland using radio waves and magnetic fields by changing the alignment of hydrogen nuclei. Unlike other scanning techniques, the MRI-scan process is complicated because fat must be suppressed. To prevent this problem, a contrasting dye or drug—usually based on gadolinium, is used to distinguish it from other tissues. The main disadvantages of using MRI for thyroid scanning include the high cost of operating the machine, the cost of having trained experts and professional radiologists interpret the images, and its relatively low specificity [3].

Ultrasonography is another non-invasive method used to detect thyroid cancer; it creates images of nodular masses using sound waves. The technician rotates a hand-held transducer on the surface of the skin to generate an image of what is directly underneath it. The use of B-mode ultrasound has high sensitivity in distinguishing malignant tumors and shows low-echo, microcalcification, hypervascularity, and irregular borders. Tumors showing more blood distribution in the central area than marginal areas on the Doppler ultrasound have a high likelihood of malignancy. One disadvantage of this method is that individual images must be labeled by the technician performing the tests to generate each map. Moreover, interferences, such as speckles, often blur the image, lateral margins of lesions are difficult to detect, and ultrasonography generally suffers from low contrast.

The limitations of ultrasound are being overcome with the recent introduction of a new imaging technology—sonoelastography. Non-invasive sonoelastography measures hardness and captures real-time images of nodular tissues to determine the degree of elasticity—relying on malignant tissues being more rigid than normal or benign tissues. While tissues are vibrated at a low frequency, the sonography technique examines their relative stiffness or elasticity. The use of elastography techniques for the non-invasive evaluation of tissues’ mechanical properties has attracted substantial interest in recent years. These techniques utilize elasticity changes in soft tissues in various pathologies to produce qualitative and quantitative information that can be used for diagnosis. Improved ultrasound-based methods are drawing increasing attention because of their several inherent advantages, such as their wide availability (including bedside availability), and relatively low cost. In particular, solid tumors are known to be mechanically different from surrounding healthy tissues. Thus, it is crucial to acquire the ability to investigate changes in tissue elasticity that may indicate calcifications or actual tumors without exposing patients to radiation.

In addition to sonoelastography, a photoacoustic imaging method that obtains ultrasound signals using light has also been introduced. When light shines on the patient’s thyroid nodule, ultrasound signals are generated from the thyroid and nodule. The photoacoustic images of the thyroid gland and surrounding nodules can be obtained by acquiring and processing these signals. Moreover, by acquiring photoacoustic signals using light of various colors, information on the oxygen saturation in the thyroid gland and nodules can be obtained. This information is analyzed using a machine-learning technique to automatically classify thyroid nodules as malignant or benign.

The TSIS is a type of elasticity-imaging system. Elasticity imaging is also known as stress, mechanical, tactile, elastic-modulus, and biomechanical imaging. Stress imaging [4] is under extensive investigation by many researchers. Strain-imaging techniques using other sensors, such as piezoelectric cantilevers, also exist, but require an electronic circuit board and extensive calibration.

Tactile sensors have recently become common in diagnostic and surgical procedures. In minimally invasive surgery (MIS), medical personnel cannot obtain tactile information by directly palpating the cancer site [5,6]. Thus, a diagnosis is made by attaching a tactile sensor integrated with an injection needle for a robot endoscope that can be introduced into a surgical instrument. The tactile sensor can detect the hardness of tissues by changing the resonance frequency based on the piezoelectric effect. Moreover, an autonomous palpation algorithm was developed to accurately locate tumors and identify their boundaries. The stimulation of tactile systems varies according to the efficiency of tactility-perception strategies, the technical aspects of sensory devices, and the design of effective tactile-learning methods. Current systems can be improved by leveraging advances in signal processing and fabrication techniques, such as the use of highly flexible and elastic materials, including polydimethylsiloxane (PDMS), carbon nanotubes, biophoretic materials, and graphene. The use of these microfabrication techniques and integrated materials is intended to develop prosthetic skins with built-in tactile sensing capability, which will play a critical role in multifunctional electronic skins, hand prostheses, and soft robots in the future.

**Figure 1:**
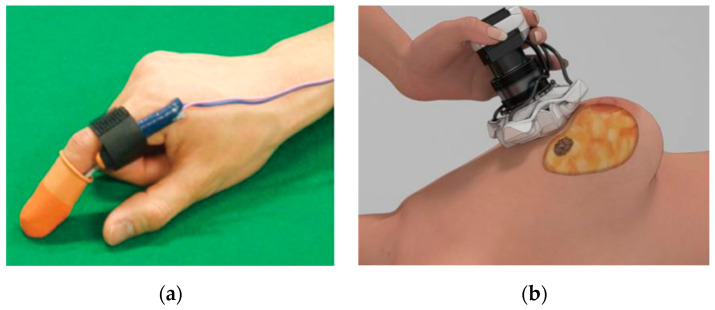
(**a**) Prostate-cancer diagnosis based on tactile sensation “Reproduced with permission from [Ahn, B.; Park, K.; Lee, H.; Kim, J…], [A Survey of Robotic Technologies for Diagnosis and Treatment of Prostate Cancer.]; published by [Control, Robotics and Systems], [2010].” [8]. (**b**) Breast-cancer diagnosis based on optical tactile elasticity “Reproduced with permission from [Yang, S.; Lee, J.-H.], [Tissue Hardness Measurement Sensor and Algorithm for Tumor Detection Using Light Trapping and Scattering in Silicone Waveguide]; published by [Sensors and Materials], [2018].” [9].

Recently, cancer diagnosis using tactile sensation has been extensively researched [1,5,6]. Research on robot palpation systems [7] that derive precise and quantitative diagnosis results has enabled accurate diagnoses by overcoming the limitations of subjective diagnoses through digital rectal examinations conducted using conventional tactile-diagnosis methods. This new method can reduce differences in diagnoses based on palpation according to the proficiency of the operator and can provide objective results through quantification by assisting the subjective judgment of the doctor. A common breast-cancer-palpation method is to rub the breast with the hands and search for hardened sites. However, this palpation method is subjective and the recording of tactile sensations is difficult. In order to increase the accuracy, an algorithm for diagnosing and predicting breast cancer based on fusion images has also been researched. In this method, a tactile and a near-infrared image are matched by acquiring them together. Figure 1a shows a system for diagnosing prostate cancer with a tactile-based robot palpation system, and Figure 1b presents a system for diagnosing breast cancer based on optical tactile elasticity.

The current trend is the development of technology to miniaturize tactile-sensor arrays and create a flexible tactile-sensing system that can measure adhesive and shear forces simultaneously. These tactile sensors are expected to enjoy a wide range of applications in many fields in the future. Science and engineering have developed beyond the human instinct to see and observe things, to touch and accept them within the mind. These human instincts are expected to accelerate the development of tactile sensors.

A sensor was recently introduced to detect tumor progression and regression by measuring tumor volume. It is composed of a flexible and stretchable skin-like polymer that includes an embedded layer of gold circuitry. It is a small, autonomous device with a stretchable and flexible sensor that can be adhered to the skin to measure the changing sizes of tumors below. This non-invasive, battery-operated device is sensitive to one-hundredth of a millimeter (10 μm) and can beam results to a smartphone app wirelessly in real time with the pressing of a button [10].

Tactile sensations that can be felt with the human skin, hands, and feet can be utilized for every object. Thus, it is difficult to discuss tactile sensations without mentioning the connection between engineering and future society. When an object is touched, the human mind understands and feels it instantaneously. Therefore, tactile-sensor technology has near-infinite possibilities for development.

### 1.2. Contributions of This Study

The objective of this study is to develop a simple and easy-to-use tactile-sensation-imaging system that documents tissue properties using a non-invasive technique. In order to build a digitalized self-examination system, the system’s construction and examination cost must be low, there must be no radiation exposure, and examination should be easy, without the help of a doctor. Biomechanically, thyroid lesions exhibit inconsistent elasticity due to interstitial collagen and flakes—thus, they are influenced by the stiffness of healthy cells and, therefore, by tactile sensations.

The proposed tactile sensor consists of an elastic optical waveguide, a high-resolution CCD camera, a LED light source, and a laptop computer. The sensing element is embedded in the PDMS along four sides by LED light sources. Tactile sensors work on the principle of total internal reflection (TIR) inside an optical waveguide. Directing light to the waveguide is a simple way to obtain a tactile image using the principle of TIR in silicon. As the system probes the hardened part, deformation occurs, and the light that was previously totally reflected becomes diffusely reflected and is captured as a tactile image. The method of accurately recording the tactile sensations obtained through patients will improve the ability to detect changes in mass over time.

**Figure 2:**
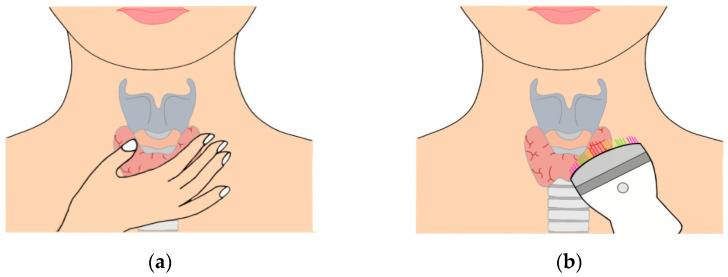
(**a**) Thyroid self-diagnosis. (**b**) Tactile recording system using optical tactile-elasticity imaging.

This study intends to provide a solution to the problem of digitizing tactile imaging results. The proposed method provides a cheaper and reproducible method for estimating the size of nodules compared to more widely used thyroid-cancer-screening techniques, such as ultrasonography and thyroid imaging. Figure 2a shows tactile diagnosis, a current thyroid self-diagnosis method, and Figure 2b shows the proposed tactile-diagnosis system based on optical tactile-elasticity imaging. This quantifies the sensation of the hand with the touch of the tactile-imaging system.

## 2. Research Subjects and Method

### 2.1. Tactile-Imaging Sensor

The basic design of tactile sensor is introduced to satisfy the technical requirements of this study based on the specific characteristics of human fingers. The tactile sensors based on the structure of human fingers were designed with the following characteristics.

(1) PDMS: PDMS, which imitates human tissue, can effectively detect the texture of soft materials and is necessary to provide smooth contact surfaces.
(2) Three-layer structure: Three types of PDMS with different levels of elasticity are stacked onto each other to imitate the natural human-tissue structure. The elasticity of PDMS is specified by its catalyst ratio.
(3) Bone and nail elements: The elements that function as bones and nails are positioned underneath the sensor in order to effectively derive sensory information. This design utilizes a heat-resistant borosilicate glass plate.
(4) Distributed sensor elements: An optical method that imitates the mechanoreceptors of the finger and uses a light-reflection pattern is adopted to obtain the spatial distribution of the sensory output.

### 2.2. System Figures and Tables

**Figure 3:**
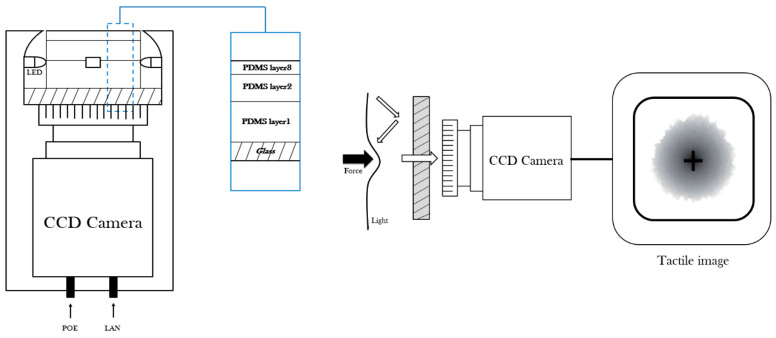
Schematic of the optical tactile sensor.

Figure 3 shows the structure of the proposed tactile sensor. This structure is composed of an elastic optical waveguide unit, a camera unit, and a computer for data analysis. The optical waveguide is illuminated by four white LED light-source units attached to a glass plate.

#### 2.2.1. Elastic Optical Waveguide

**Figure 4:**
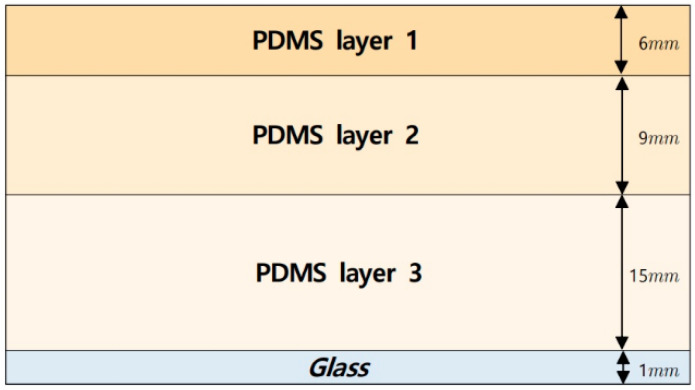
Structure of the multilayer optical waveguide.

The elastic optical waveguide is the main sensing probe of the device and is composed of PDMS (high-performance silicon). The PDMS must satisfy the requirements for a transparent optical waveguide. Figure 4 illustrates a schematic of the multilayer elastic waveguide. The heat-resistant borosilicate glass plate should be perfectly horizontal and placed over the PDMS.

#### 2.2.2. Near-Infrared Camera

A near-infrared camera is positioned beneath the optical waveguide. Transparent glass is placed between the camera and optical waveguide to maintain elasticity of optical waveguide without losing camera resolution. The glass also serves as the bone and nail in human fingers. The maximum lens resolution is 656 (H) × 492 (V) and the digital image has an individual pixel size of 7.4 µm × 7.4 µm.

#### 2.2.3. Internal LED Light Source

**Figure 5:**
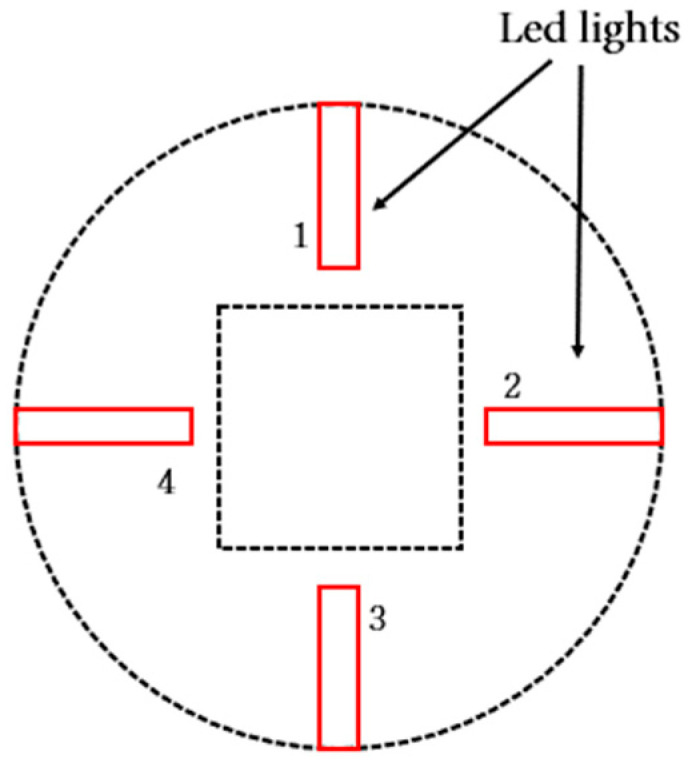
LED lights (top view).

The internal light sources are LEDs with a diameter of 5 mm. Figure 5 shows a top view of the optical waveguide depicting the location of the LEDs. The four LEDs, one on each of the four sides of the optical waveguide, provide sufficient illumination. The internal light source uses a variable resistor to adjust the brightness according to the user. Fine heat is generated while the four LEDs emit light. However, even when heat from light sources within TSIS is generated, the results of the study can clearly determine the tumor and are not an important factor. Further, even if the object in contact with the TSIS generates heat, the spread of the light is photographed by the camera, so it is not affected.

#### 2.2.4. Allied Camera with MATLAB

In order to develop a thyroid-palpation software program, the G-032 camera and MATLAB are combined to implement an application software without space constraints. The program is designed to measure the elasticity in real time by controlling the brightness and exposure of the camera within the app and to visualize the ganglion using the Open CV library. Here, a grayscale (instead of RGB) camera is used for G-032 to maximize the difference between the presence or absence of a ganglion. Images can be acquired using Image Acquisition Explorer, an app that allows users to set acquisition parameters, preview images, and acquire image data in MATLAB. After G-032 is selected from the list of devices that can be connected to the computer and app, the appropriate support package is installed. Next, it is applied in the app so that the image can be viewed in real time on the image-acquisition device using the preview and switch options. The switch is used to turn the preview on and off. The device’s properties can be changed to update the preview in real time, and the area below the preview displays the current frame rate and timestamp. The tumor area appears white, while the other areas appear gray under the internal light source controlled by the variable resistance and the refractive index. Tumors can be explored with optical imaging, and the images may be colored for a more intuitive display. Images can be visualized and analyzed by exporting the most recently saved image and saving the image data as a file or workspace variable.

#### 2.2.5. Implementation of Optical Part and Circuit Prototype of Thyroid-Tactile-Sensation-Diagnosis System

A prototype of the thyroid tactile-sensation-diagnosis system is presented in this study. For the internal light source, a 5-mm-diameter microLED is used to provide sufficient illumination for the optical waveguide. The module is designed to accommodate four LEDs. Light emitted from the light source to the edge of the optical waveguide is completely enclosed and reflected inside the optical waveguide. Tactile sensors use the deformable properties of waveguides in the conversion process. The innermost part of the waveguide transmits the light and is injected into the waveguide from the edge. At the same time, it reflects light within the boundaries of the silicon to prevent light from leaking out. When an object compresses the waveguide, the contact area of the waveguide is deformed, causing light to be scattered. Subsequently, the scattered light is captured by the CCD camera.

To control the optical part (four LEDs), a circuit consisting of a battery, switch, resistor, variable resistor, jumper cable, and mini breadboard was designed, and its operation was verified. The device was designed for permanent use by using a rechargeable battery that controls the LED power by using a switch. This circuit employs a variable resistor and the intensity of the LED light can be adjusted by turning the sweeper of the variable resistor by adjusting the size of the current. The LED is miniaturized by connecting the battery’s positive (+) and negative (−) poles to a mini breadboard. The positive (+) and negative (−) poles of the mini breadboard and the LED are connected in series to the battery’s positive (+) and negative (−) poles using jumper cables. After connecting them with all the LEDs, the resistor, variable resistor, and switch are connected to the breadboard. Sufficient illumination of the optical waveguide can be ensured by controlling the optics.

## 3. Research Details and Results

### 3.1. System Design and Imaging Principle

#### 3.1.1. Optical-Waveguide Design

A waveguide is a hollow metal tube used to transmit electrical signals or energy with frequencies higher than those of microwaves. The cross-section of a waveguide is usually rectangular or circular, and only signals with the same wavelength as the cross-section can pass through the waveguide. An optical waveguide, which is a tube using a light-transmitting material, was used as the primary sensing probe of the system in this study. This waveguide was composed of PDMS, a high-performance silicon elastomer, because it needed flowability, elasticity, and transparency.

We imitated the tissue structure of the human finger to achieve human tactile levels. Human-finger tissue is divided into three layers—each with a distinct modulus of elasticity. The epidermis, dermis, and subcutaneous layer make up these three layers. The epidermis is the hardest layer, with the lowest modulus of elasticity, and is approximately 1 mm thick. The dermis is a softer layer that ranges in thickness from 1–3 mm. The subcutaneous layer is the softest layer, between the dermis and the bone. The subcutaneous layer is mostly made up of fat, which serves as a cushioning substance when stresses are applied to the surface. When a finger pushes on tan item, the inner layer deforms more than the outermost layer due to the variation in the hardness of each layer. Three PDMS layers with varying moduli of elasticity were stacked together to imitate this structure. The first layer of PDMS is the hardest, followed by PDMS layer 2, with medium hardness, which is followed in turn by PDMS layer 3, which has the lowest stiffness. The ratio of the silicone elastomer base to the silicone elastomer curing agent determines the elasticity of the PDMS. The heights are roughly 6 mm for PDMS layer 1, 9 mm for PDMS layer 2, and 15 mm for PDMS layer 3. The camera is positioned underneath the optical waveguide, with a heat-resistant borosilicate glass plate between the camera and the waveguide to sustain the optical waveguide without sacrificing the resolution. The direction and angle of the light incidence from the internal light source is positioned such that it is reflected on the waveguide.

We compared PDMS using three types of silicon from two manufacturers. The elasticity variability in Case 2 was the highest, and the fabricability with elasticity variation is also included. We finally decided on Case 2, which perfectly satisfied both conditions in Table 1.

#### 3.1.2. Imaging Principle

The TIR principle was applied to the proposed system. According to Snell’s law, when light hits two media with differing refractive indices, part of the light is transmitted, and the remainder is reflected. The TIR occurs when the angle of incidence exceeds the critical angle.

**Figure 6:**
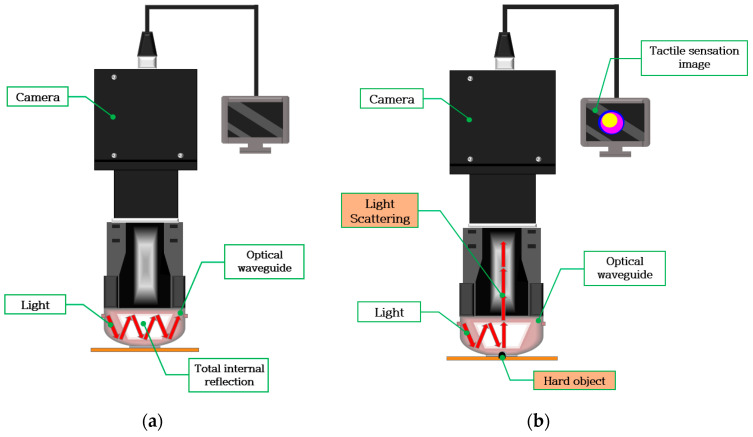
Conceptual diagram of the imaging principle. (**a**) Light is injected into the waveguide and fully reflected. (**b**) Light is scattered as the waveguide is deformed by an external force caused by a hard object.

**Figure 7:**
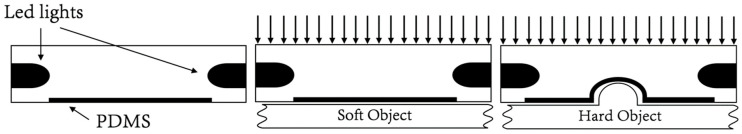
Deformation of PDMS when the waveguide is penetrated by a hard object.

The waveguide is surrounded by air in the system design in this study. Furthermore, the incident light moving toward the waveguide is completely reflected by the waveguide because the refractive index is lower than that of the PDMS layer. Waveguides are soft and elastic. Therefore, when the waveguide is compressed by the external force of a hard object, the contact area of the waveguide is deformed, and light is scattered. The scattered light is captured by a high-resolution camera and stored in an image format. The basic principle of tactile imaging is to monitor the scattering of light that occurs when the critical angle is changed by an external force. Figure 6 presents a conceptual diagram of the imaging principle. Figure 7 presents a deformation of the PDMS when the waveguide is penetrated by a hard object. The harder the object, the higher the degree of the deformation of the waveguide.

#### 3.1.3. Optical Analysis of Imaging Principle

The optical analysis of the Layer 1 waveguide was performed in the optical communication area because wave-optic analysis was performed to examine the imaging principle. This study separated the waveguide into four waveguide layers, of which three were PDMS and one was the glass plate. An analytical solution to the imaging principle was developed in the Layer 4 waveguide and presented below. Next, the numerical-simulation results of the analytical modeling are provided.

### 3.2. Analytical Solution

**Figure 8:**
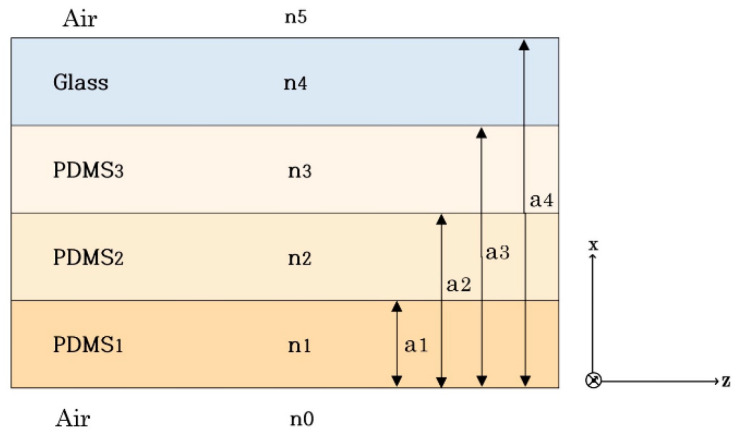
Structure of the proposed multilayer optical waveguide.

The imaging principle is defined using the wave-optics-analysis method. Figure 8 illustrates an optical waveguide comprised of three PDMS layers and one glass-plate layer.

(1) Layer 1: PDMS, refractive index n_1_, height h_1_;
(2) Layer 2: PDMS, refractive index n_2_, height h_2_;
(3) Layer 3: PDMS, refractive index n_3_, height h_3_;
(4) Layer 4: glass plate, refractive index n_4_, height h_4._

The refractive indices n_0_ and n_5_ are those of the medium that surrounds the waveguide. In this case, the refractive index of air is n_0_ = n_5_ =1. The waveguide layers are arranged in increasing order of refractive indices, n_1_ > n_2_ > n_3_ > n_4_ > n_0_ = n_5_. The layers are arranged in the x direction, whereas light propagates in the z direction. Maxwell’s wave equation describes light propagation in an optical waveguide.

The light propagation of an optical waveguide by Maxwell’s equation can be expressed as follows:

(1) $$\nabla^{2}E(x,y,z,t)-[\frac{n^{2}}{c^{2}}]\partial^{2}\frac{E(x,y,z,t)}{\partial t^{2}}=0.$$

where **E**(*x, y, z, t*) denotes the electric field, *n* denotes the refractive index, and *c* denotes the speed of light in the vacuum.

A similar equation is formed for the electromagnetic field H(*x, y, z, t*):

(2) $$\nabla^{2}E(x,y,z,t)-[\frac{n^{2}}{c^{2}}]\partial^{2}\frac{E(x,y,z,t)}{\partial t^{2}}=0.$$

We focus primarily on the electric field because the components of the electric and magnetic fields can be determined by each other. In the case of a monochromatic wave of frequency α, Equation (1) takes the following form [9]:

(x, y, z, t) = **E**(x, y, z) exp(iωt). (3)

**E**, the shape given in Equation (1), is used in Equation (3), and the spatial distribution of the electric field **E**(x, y, z) is as follows:

(4) $$\frac{\partial^{2}E(x,y,z)}{{\partial x}^{2}}+\frac{\partial^{2}E(x,y,z)}{{\partial y}^{2}}+\frac{\partial^{2}E(x,y,z)}{{\partial z}^{2}}+k_{0}^{2}n^{2}E(x,y,z)=0$$

where *k*_0_ denotes the wave vector in the vacuum: *k*_0_ = μ/c. Since the waveguide is uniform in the z direction, the plane-wave solution can be found as follows:

**E**(x, y, z) = **E**(x, y) exp(−iβz) (5)

where β is the propagation constant, which changes depending on the x direction because only the plane wave solution that is independent of the y direction is searched. Considering this, the spatial distribution of the electric field is as follows:

**E**(x, y) = **E**(x). (6)

If we assume the following to determine a solution for the y component of the magnetic field,

**E**(x) = e(x)**j**, (7)

where **j** is the unit vector of the y direction, when Equation (5) is substituted into Equation (4), it can be reduced to the following ordinary differential equation:

(8) $$\frac{d^{2}e(x)}{dx^{2}}+[k_{0}^{2}n^{2}-\beta^{2}]e(x)=0.$$

This equation must be valid in all the regions, including the waveguide and air.

(9) $$\frac{d^{2}e(x)}{dx}+[k_{0}^{2}n_{0}^{2}-\beta^{2}]e(x)=0,\;\mathrm{Region}\;0:\;x<0$$

(10) $$\frac{d^{2}e(x)}{dx}+[k_{0}^{2}n_{1}^{2}-\beta^{2}]e(x)=0,\;\mathrm{Region}\;1:\;0<x<a1$$

(11) $$\frac{d^{2}e(x)}{dx}+[k_{0}^{2}n_{2}^{2}-\beta^{2}]e(x)=0,\;\mathrm{Region}\;2:\;a1<x<a2$$

(12) $$\frac{d^{2}e(x)}{dx}+[k_{0}^{2}n_{3}^{2}-\beta^{2}]e(x)=0,\;\mathrm{Region}\;3:\;a2<x<a3$$

(13) $$\frac{d^{2}e(x)}{dx}+[k_{0}^{2}n_{3}^{2}-\beta^{2}]e(x)=0,\;\mathrm{Region}\;4:\;a3<x<a4$$

(14) $$\frac{d^{2}e(x)}{dx}+[k_{0}^{2}n_{4}^{2}-\beta^{2}]e(x)=0,\;\mathrm{Region}\;5:\;x>a4$$

Here, regions 0 and 5 are outside the waveguide, whereas regions 1–4 are inside each waveguide layer. As light does not propagate outside the waveguide, the assumed solutions of regions 0 and 5 must decrease exponentially with the distance from the surface. The propagating light in regions 1–4 vibrates and has a sinusoidal wave form. Therefore, we assume the form of the solution from (9) to (14) as follows:

(15) $$e(x)=e_{0}\;\exp[k_{0}x],\;\mathrm{Region}\;0:\;x<0$$

(16) $$e(x)=e_{1}\;\cos[k_{1}x+\varphi_{1}],\;\mathrm{Region}\;1:\;0<x<a1$$

(17) $$e(x)=e_{2}\;\cos[k_{2}x+\varphi_{2}],\;\mathrm{Region}\;2:\;a1<x<a2$$

(18) $$e(x)=e_{3}\;\cos[k_{3}x+\varphi_{3}],\;\mathrm{Region}\;3:\;a2<x<a3$$

(19) $$e(x)=e_{4}\;\cos[k_{4}x+\varphi_{4}],\;\mathrm{Region}\;4:\;a3<x<a4$$

(20) $$e(x)=e_{5}\;\exp[k_{5}(a4-x)].\;\mathrm{Region}\;5:\;x>a4$$

The solution is determined by using unknown parameters, such as amplitude e_i_, transverse wave vector φ_i_, and phase φ_i_, where i = 0, 1, 2, 3, 4, 5. These parameters must be determined from the boundary conditions matching the fields of different regions. These solutions are substituted in each equation from (15) to (20). When we examine the dispersion relation up to (14),

(21) $$-k_{0}^{2}+\beta^{2}=k_{0}^{2}n_{0}^{2}\;\mathrm{Region}\;0:\;x<0$$

(22) $$k_{1}^{2}+\beta^{2}=k_{0}^{2}n_{1}^{2}\;\mathrm{Region}\;1:\;a0<x<a1$$

(23) $$k_{2}^{2}+\beta^{2}=k_{0}^{2}n_{2}^{2}\;\mathrm{Region}\;2:\;a1<x<a2$$

(24) $$k_{3}^{2}+\beta^{2}=k_{0}^{2}n_{3}^{2}\;\mathrm{Region}\;3:\;a2<x<a3$$

(25) $$k_{4}^{2}+\beta^{2}=k_{0}^{2}n_{4}^{2}\;\mathrm{Region}\;4:\;a3<x<a4$$

(26) $$-k_{5}^{2}+\beta^{2}=k_{0}^{2}n_{5}^{2}\;\mathrm{Region}\;5:x>a4$$

Furthermore, boundary conditions must be applied, and field components must be matched. To this end, the magnetic field must be determined first from Maxwell’s equation. The magnetic field has a shape similar to the electric field, but now has only one non-zero component along the z direction, as follows:

(27) H (x, y, z)=kh(x) exp(−iβz+iωt),

where k is the unit vector in the z direction. When Equations (5) and (27) are substituted into Maxwell’s equation,

(28) $$\nabla \;\times \;E=-\frac{1}{\left(c\right)},\frac{\partial H}{\partial t}$$

For the magnetic fields expressed through parameters such as the electric field, the following general solutions can be obtained:

(29) $$h(x)=-(\frac{ic}{w})k_{0}e_{0}\exp[k_{0}x],\;\mathrm{Region}\;0:\;x<0$$

(30) $$h(x)=(\frac{ic}{w}){k_{1}e}_{1}\sin[k_{1}x+\varphi_{1}],\;\mathrm{Region}\;1:\;0<x<a1$$

(31) $$h(x)=(\frac{ic}{w}){k_{2}e}_{2}\sin[k_{2}x+\varphi_{2}],\;\mathrm{Region}\;2:\;a1<x<a2$$

(32) $$h(x)=(\frac{ic}{w}){k_{3}e}_{3}\sin[k_{3}x+\varphi_{3}],\;\mathrm{Region}\;3:\;a2<x<a3$$

(33) $$h(x)=(\frac{ic}{w}){k_{4}e}_{4}\sin[k_{4}x+\varphi_{4}],\;\mathrm{Region}\;4:\;a3<x<a4$$

(34) $$h(x)=(\frac{ic}{w}){k_{5}e}_{5}\exp[k_{5}(a4-x)],\;\mathrm{Region}\;5:\;x>a4$$

The ratio of h(x) to e(x), which are the amplitudes of the electric and magnetic fields, respectively, is called impedance. The impedance h(x)/e(x) must be continuous at all boundaries in x = 0, x = a_1_, x = a_2_, x = a_3_, and x = a_4_, as follows:

(35) $$-\left(\frac{ic}{w}\right)\frac{k_{0}e_{0}\exp\left[k_{0}x\right]}{e_{0}\exp\left[k_{0}x\right]}=\left(\frac{ic}{w}\right)\frac{k_{1}e_{1}\sin\left[k_{1}x+\emptyset_{1}\right]}{e_{1}\cos\left[k_{1}x+\emptyset_{1}\right]},\;\mathrm{Boundary}\;1:\;x=0,$$

(36) $$\left(\frac{ic}{w}\right)\frac{k_{1}e_{1}\sin\left[k_{1}x+\emptyset_{1}\right]}{e_{1}\cos\left[k_{1}x+\emptyset_{1}\right]}=\left(\frac{ic}{w}\right)\frac{k_{2}e_{2}\sin\left[k_{2}x+\emptyset_{2}\right]}{e_{2}\cos\left[k_{2}x+\emptyset_{2}\right]},\;\mathrm{Boundary}\;2:\;x=a1$$

(37) $$\left(\frac{ic}{w}\right)\frac{k_{2}e_{2}\sin\left[k_{2}x+\emptyset_{2}\right]}{e_{2}\cos\left[k_{2}x+\emptyset_{2}\right]}=\left(\frac{ic}{w}\right)\frac{k_{3}e_{3}\sin\left[k_{3}x+\emptyset_{3}\right]}{e_{3}\cos\left[k_{3}x+\emptyset_{3}\right]},\;\mathrm{Boundary}\;3:\;x=a2$$

(38) $$\left(\frac{ic}{w}\right)\frac{k_{3}e_{3}\sin\left[k_{3}x+\emptyset_{3}\right]}{e_{3}\cos\left[k_{3}x+\emptyset_{3}\right]}=\left(\frac{ic}{w}\right)\frac{k_{4}e_{4}\sin\left[k_{4}x+\emptyset_{4}\right]}{e_{4}\cos\left[k_{4}x+\emptyset_{4}\right]},\;\mathrm{Boundary}\;4:\;x=a3$$

(39) $$\left(\frac{ic}{w}\right)\frac{k_{4}e_{4}\sin\left[k_{4}x+\emptyset_{4}\right]}{e_{4}\cos\left[k_{4}x+\emptyset_{4}\right]}=\left(\frac{ic}{w}\right)\frac{k_{5}e_{5}\exp\left[k_{5}(a_{4}-x)\right]}{e_{5}\exp\left[k_{5}(a_{4}-x)\right]},\;\mathrm{Boundary}\;5:\;x=a4$$

The following equations can be obtained:

(40) $$k_{0}=-k_{1}\tan(\varphi_{1}),\;\mathrm{Boundary}\;1:\;x=0$$

(41) $$k_{1}\tan(k_{1}a_{1}+\varphi_{1})=-k_{2}\tan(k_{2}a_{1}+\varphi_{2}),\;\mathrm{Boundary}\;2:\;x=a1$$

(42) $$k_{2}\tan(k_{2}a_{2}+\varphi_{2})=-k_{3}\tan(k_{3}a_{2}+\varphi_{3}),\;\mathrm{Boundary}\;3:\;x=a2$$

(43) $$k_{3}\tan(k_{3}a_{3}+\varphi_{3})=-k_{4}\tan(k_{4}a_{3}+\varphi_{4}),\;\mathrm{Boundary}\;4:\;x=a3$$

(44) $$k_{4}\tan(k_{4}a_{4}+\varphi_{4})=-k_{5},\;\mathrm{Boundary}\;5:\;x=a4$$

The above equations can be substituted as follows:

(45) $$\phi_{1}=-arctan(\frac{k_{0}}{k_{1}}),$$

(46) $$\phi_{2}=\arctan[(\frac{k_{1}}{k_{2}})\;\tan(k_{1}a_{1}+\varphi_{1})]\;-k_{2}a_{2},$$

(47) $$\phi_{3}=\arctan[(\frac{k_{2}}{k_{3}})\;\tan(k_{4}a_{3}+\varphi_{4})]-k_{3}a_{3},$$

(48) $$\phi_{4}=\arctan(\frac{k_{5}}{k_{4}})-k_{4}a_{4},$$

(49) $$k_{0}=\sqrt{\beta^{2}-k_{0}^{2}n_{0}^{1}},$$

(50) $$k_{1}=\sqrt{k_{0}^{2}n_{1}^{2}{-\beta }^{2}},$$

(51) $$k_{2}=\sqrt{k_{0}^{2}n_{2}^{2}{-\beta }^{2}},$$

(52) $$k_{3}=\sqrt{k_{0}^{2}n_{3}^{2}{-\beta }^{2}},$$

(53) $$k_{4}=\sqrt{k_{0}^{2}n_{4}^{2}{-\beta }^{2}},$$

(54) $$k_{5}=\sqrt{\beta^{2}-k_{5}^{2}n_{5}^{2}},$$

The amplitude is also determined from the boundary conditions:

(55) $${e_{1}=e}_{0}\cos(\emptyset_{1}),\;\mathrm{Boundary}\;1:\;x=0$$

(56) $${e_{2}=e}_{1}\frac{\cos(k_{1}a_{1}+\emptyset_{1})}{\cos(k_{2}a_{1}+\emptyset_{2})},\;\mathrm{Boundary}\;2:\;x=a1$$

(57) $$e_{3}=e_{2}\frac{\cos(k_{2}a_{2}+\emptyset_{2})}{\cos(k_{3}a_{2}+\emptyset_{3})},\;\mathrm{Boundary}\;3:\;x=a2$$

(58) $${e_{4}=e}_{3}\frac{\cos(k_{3}a_{3}+\emptyset_{3})}{\cos(k_{4}a_{3}+\emptyset_{4})},\;\mathrm{Boundary}\;4:\;x=a3$$

(59) $${e_{5}=e}_{4}\cos(\cos(k_{4}a_{4}+\emptyset_{4}),\;\mathrm{Boundary}\;5:\;x=a4$$

After replacing Equations (45)–(54) with Equations (40)–(44), the only variable is the propagation constant α. Light is a kind of electromagnetic wave. Electromagnetic waves propagate in the form of two vector waves coupled together: electric field wave, E, and magnetic field wave, H. Thus, the solutions to Equations (40)–(44) provide the whole spectrum of light propagation in the waveguide.

### 3.3. Numeric Simulations

**Figure 9:**
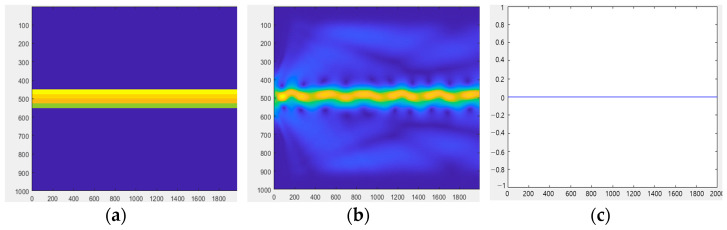
(**a**) Optical waveguide viewed from the side. (**b**) Scattering in the optical waveguide. (**c**) Optical waveguide.

Equations (40)–(44) are used to quantitatively simulate the imaging principle. It is feasible to obtain the electromagnetic wave pattern in the multilayer optical waveguide and demonstrate TIR using a numerical simulation. It is also proved that when an external force deforms the optical waveguide, light is dispersed and visible on the surface of the optical waveguide. Wave optic analysis is too complex for thick waveguides. As a result, the waveguide is considered to be very narrow in this simulation. Wavelengths that are a few millimeters thick must be approximated by light beams using a geometrical optical approximation technique. Figure 9 illustrates an optical waveguide viewed from the side.

Figure 9a shows the optical waveguide before light injection viewed from the side. The three PDMS layers and one glass-plate layer are indicated by different colors. We assume that the light is injected from the waveguide’s left side.

Figure 9b shows the result of the light injection. A tiny fraction of the light injected into the waveguide is diffracted, owing to discontinuities in the medium, air, and waveguide. The image also captures the light scattered from the top surface of the optical waveguide, and Figure 9c displays the result. Figure 9c shows that the ray without light scattering converges to 0, since the light is totally reflected by the optical waveguide.

**Figure 10:**
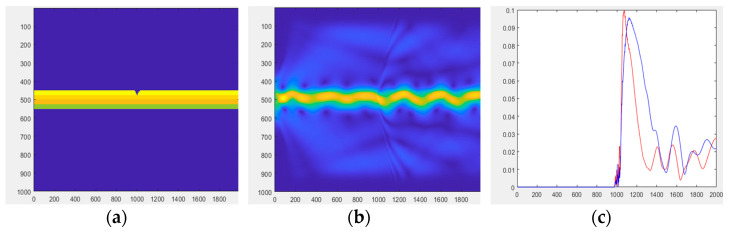
(**a**) Deformed optical waveguide viewed from the side. (**b**) Scattering inside the optical waveguide due to waveguide deformation. (**c**) Optical waveguide.

Next, Figure 10 shows the light dispersion when the waveguide is deformed. Except for a slight groove on the top surface, this waveguide is comparable to a planar waveguide, which, similar to a waveguide, permits light to travel a set distance before reaching the groove. Light must first couple with the waveguide for wave propagation in the deformed four-layer waveguide, and when the light enters into contact with the groove, additional light is scattered from the waveguide surface. Figure 10b shows the result of the light injection. Rays 1 (red) and 2 (blue) were traced from the waveguide deformation’s starting point in Figure 10c. When light is injected into the waveguide, rays 1 and 2 touch the deformed region, and scattered light is generated from the surface of the waveguide.

### 3.4. Geometric Optics Approximation

**Figure 11:**
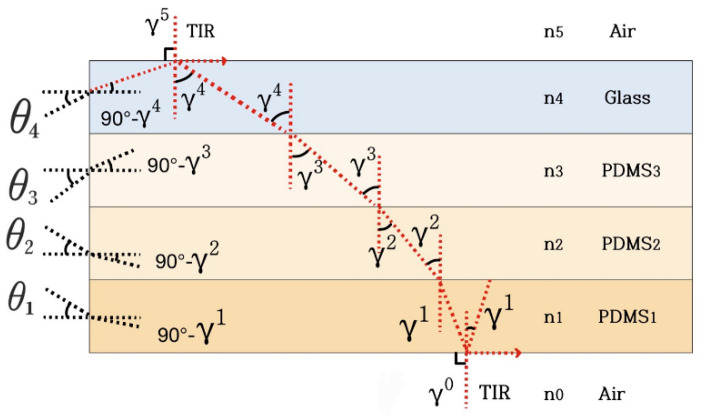
Expressions of light propagation in a waveguide at the propagation angles of θ_i_, where i = 0, 1, 2, 3, 4, 5.

Section 3.3 considered the light propagation in a waveguide to be an electromagnetic field. Figure 11 shows the boundary condition between the PDMS layers may be used to mathematically represent the solution to Maxwell’s equation. This explanation of wave propagation is called the physical optics approach, and it is appropriate for waveguides with layers as thin as a few microns. The wave-optical analysis for thin waveguides is considerably difficult when compared to thick waveguides. As a result, light propagating through a thick waveguide should be considered a ray. This method, known as geometric optical approximation, is an alternative to wave optics and may be used for thick-layered waveguides. The acceptance angle of light may be calculated using geometrical optical approximation. The acceptance angle is the largest angle that is allowed for the TIR of light. The critical and allowed angles were calculated using geometric optical approximation. Light waves were assumed to be rays in this approximation. The direction of light illumination can be determined through this method.

(60) $$n_{1}\;\sin\;\theta_{0}=n_{1}\;\sin\;\theta_{0},$$

(61) $$n_{2}\;\sin\;\theta_{2}=n_{1}\;\sin\;\theta_{1},$$

(62) $$n_{3}\;\sin\;\theta_{3}=n_{2}\;\sin\;\theta_{2},$$

(63) $$n_{4}\;\sin\;\theta_{4}=n_{3}\;\sin\;\theta_{3},$$

(64) $$n_{5}\;\sin\;\theta_{5}=n_{4}\;\sin\;\theta_{4}.$$

The TIR condition is attained when θ_0_ = θ_5_ = 90° at the waveguide–air interface. Light propagating beyond the angle θ_i_ in each layer of the waveguide is confined inside the waveguide. The critical angle denotes the smallest propagation angle θ_i_. The allowed angle θ_i_ of the incident light with respect to the waveguide is determined to make the propagation angle θ_i_ larger than the critical angle. The acceptance angle θ_i_ is the largest angle at which light directed into a waveguide stays confined. By the same Snell’s law [11], the propagation angle θ_i_ is related to the acceptance angle θ_i_.

(65) $$sin\theta_{1}=n_{i}\sin\left(90^{{}^{\circ}}-\gamma_{i}\right)=n_{i}cos\gamma_{i}$$

Moreover, Equation (65) can be rewritten as follows:

(66) $$sin\theta_{1}=n_{i}cos\gamma_{i}=n_{i}(1-{sin}^{2}\gamma_{i}{)}^{\frac{1}{2}}=(n_{i}^{2}-n_{i}^{2}{sin}^{2}\gamma_{i}{)}^{\frac{1}{2}}$$

However, from Equations (60)–(64), every n_i_ sin θ_i_ equals n_0_, which equals 1 in the case of air. Hence, the acceptance angle θ_i_ corresponds to each layer i.

(67) $$\theta_{i}=\mathrm{asin}[(n_{i}^{2}-1{)]}^{\frac{1}{2}}$$

The light incident to layer i beneath the acceptance angle θ_i_ becomes confined inside the waveguide.

The refractive index of each PDMS layer and the glass plate in the present design are approximately measured at 1.41, 1.40, 1.39, and 1.38, respectively. The allowed angles θ_i_ are calculated as θ_1_ = 83.73°, θ_2_ = 78.46°, θ_3_ = 74.89°, and θ_4_ = 71.98°. As a result, the TIR in the waveguide was positioned to inject light by choosing a spatial radiation pattern of the LED light with an angle smaller than 71.98° × 2 = 143.96°.

### 3.5. TIR-Based Tactile Sensing

**Figure 12:**
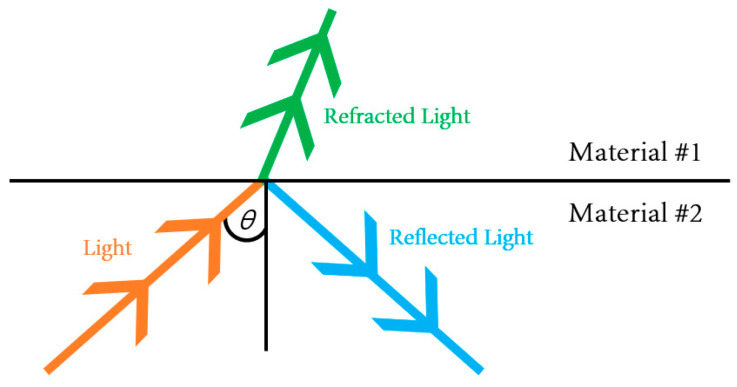
Reflection concept of the TIR principle.

This technique is the foundation of multi-touch sensing technology, which is utilized in ordinary touch screens and is based on the TIR principle. Every material, including air, water, metal, and plastic, has a refractive index. Light is either reflected or refracted as it passes from one substance to another. Figure 12 shows whether light is reflected or refracted is determined by the refractive index of the two materials and the angle at which the light emerges or enters the media.

Tactile sensors follow the TIR principle. Light emitted through the optical waveguide’s edge is entirely confined and reflected inside the optical waveguide. Tactile sensors take advantage of the waveguide’s deformable characteristic. The innermost region of the waveguide transmits and reflects light inside the silicon’s border, preventing light from leaking out. When an object collides with and compresses an optical waveguide, the deformed part scatters light.

**Figure 13:**
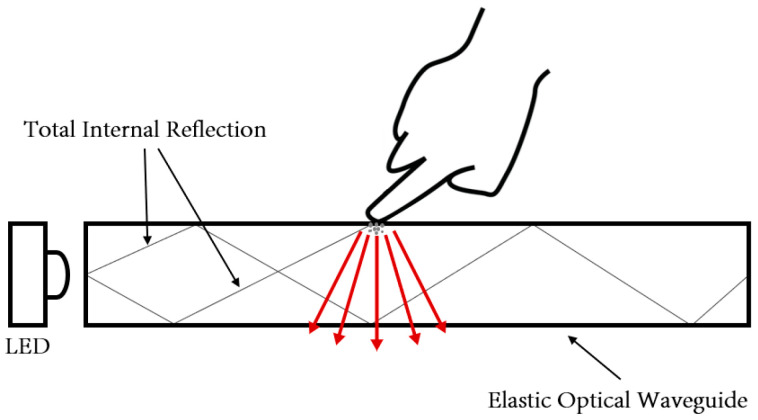
Tactility concept using the TIR principle.

The TIR is explained by Snell’s law, according to which the angle at which light is reflected when it passes from one substance to another is determined by the refractive indices of the two materials (in this case, the elastic optical waveguide and air). Each medium is characterized by a unique complex refractive index N = n − ik, where N denotes the complex refractive index, n denotes the real component, and k denotes the imaginary part of the refractive index. The extinction coefficient is often given as k. The refractive index is the ratio (n = c/v) of the speed of light (c) in a vacuum to the speed of light (v) in a certain medium (v). Light is injected into the silicon PDMS and is constantly reflected inward across the screen, effectively bouncing within the silicon PDMS. When a finger touches the silicon PDMS screen, the finger’s refractive index obstructs the light-reflection route. Figure 13 shows this light exits from the silicon PDMS and is reflected or scattered on the fingertip, where it shines.

### 3.6. Imitation Thyroid

**Figure 14:**
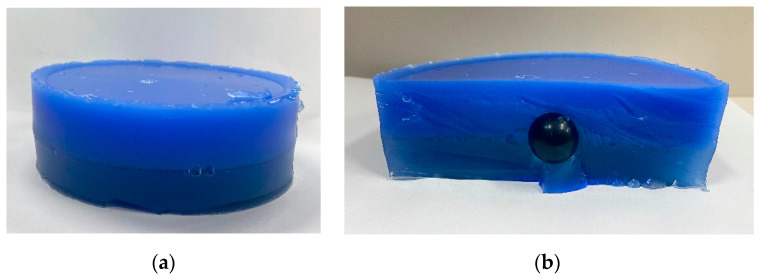
(**a**) Human phantom. (**b**) Cross-sectional view of the phantom.

In this experiment, a total of nine phantoms (with conditions comparable to those of the human body) were fabricated by modifying the depth, size, and elasticity of the nodules in only one out of the three cases. A simulation experiment was conducted to determine the presence or absence of a tumor using gelatin, the material that can produce the most similar structure and strength to the human body. Gelatin can be used as a substitute for muscle tissue in ballistic tests to produce a formulation similar to that of the human body [12]. Therefore, experiments were conducted to find the most suitable concentration, and a total of four concentrations were produced to show the hardness that was most similar to that of the human body. Therefore, all of the gelatin-model experiments were conducted using gelatin produced at the previously presented concentration. During the experiment, Figure 14 shows the ganglions were fabricated to be invisible using a blue pigment under the premise of imaging unseen nodules in a real body. When the nine tissue phantoms were imaged using an optical tactile-imaging system, distinct tactile data based on the depth, size, and elasticity were retrieved. In other words, research demonstrates that with this system, it is feasible to diagnose invisible thyroid cancer in the human body and forecast the size of the nodule based on the characteristics of ganglions.

### 3.7. Tactile-Sensation-Imaging Device

**Figure 15:**
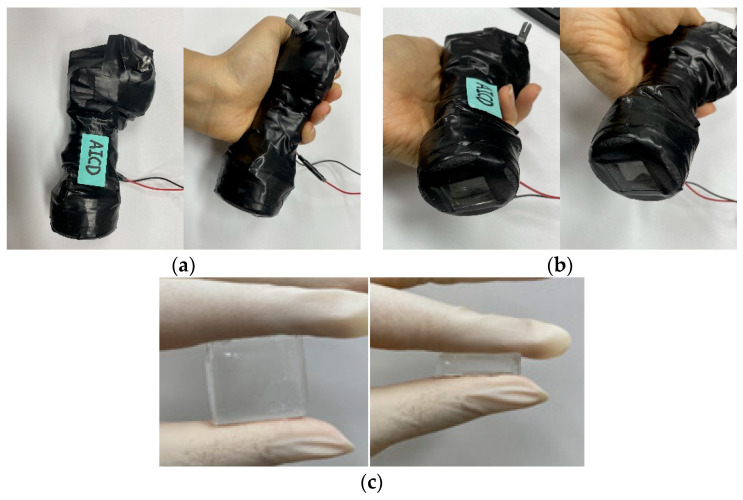
(**a**) Optical tactile-imaging device. (**b**) Bonded site of PDMS. (**c**) Final PDMS photographs.

This experiment was carried out to demonstrate that thyroid cancer may be detected using an optical tactile-imaging device. Figure 15a shows an optical tactile-imaging device that uses a CCD camera to generate a tactile image utilizing the TIR principle. This allows the identification of thyroid cancer and the estimation of nodule size. Figure 15b shows the side of the system that is combined with the blended-elasticity PDMS. The device was intended to replace the PDMS and was built in the same size as the optical waveguide. Figure 15c is the final PDMS picture, which was created with appropriate elasticity by adjusting the silicon ratio. As PDMS employs the TIR principle, it should be free of air bubbles and foreign debris and retain its transparency.

**Figure 16:**
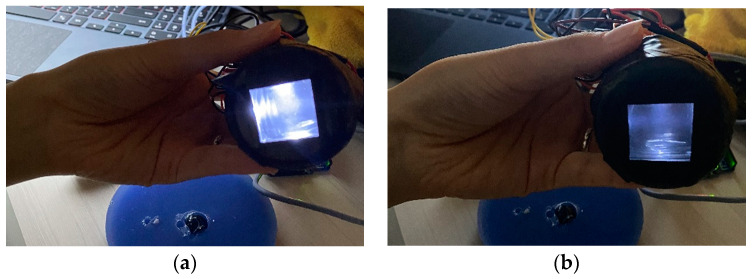
Adjusting the optical waveguide’s brightness: (**a**) maximum brightness, (**b**) medium brightness.

**Figure 17:**
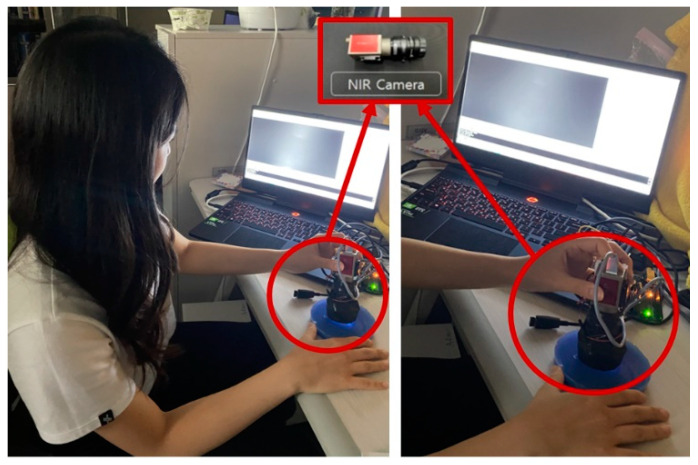
Optical tactile imaging.

Figure 16 shows the total power of the LED, built in the optical waveguide, which is controlled by a toggle switch. During diagnosis, a variable resistor is also employed to alter the degree of injected LED light. More precise and distinct results may be obtained by altering the intensity of the LED. Figure 17 shows the image taken directly using TSIS.

## 4. Discussion and Conclusions

### 4.1. Experimental Results

The diagnostic device’s ability to define the ganglions found in each tissue is shown. Tissue phantoms were created for this experiment based on elasticity, depth, and size. The phantoms were fabricated under the following conditions: the depth and size of the embedded nodule were fixed, but the elasticity was changed; the size and elasticity were fixed but the depth was changed; and the depth and elasticity were fixed but the size was changed. The phantom’s thickness was 40 mm, and the nodules were placed 5 mm below the phantom’s top surface. As light was scattered in the contact region, the grayscale value of the image acquired by the camera was distributed in a shape in which the grayscale intensity was greatest at the center and dropped as it moved away from the center of the deformation area. Colormap tactile data were produced by 2D simulation and the form of the tactile data was described using a 3D model in order to compare the tactile pictures based on elasticity. An optical image-processing algorithm was used to search for and visualize tumors, and a 3D-elasticity-image algorithm was developed to present them more intuitively.

#### 4.1.1. Inclusion-Elasticity Phantom

**Figure 18:**
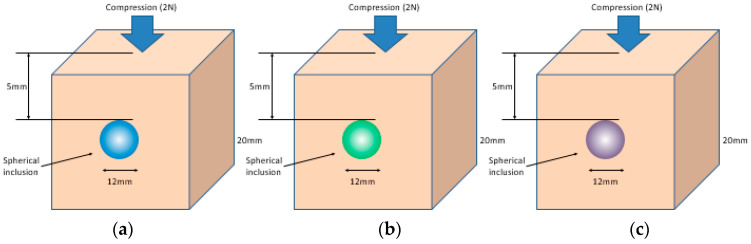
Tactile images of three different hardness values embedded in the phantom: (**a**) 110 kPa of tissue included, (**b**) 80 kPa of tissue included, and (**c**) 50 kPa of tissue included.

**Figure 19:**
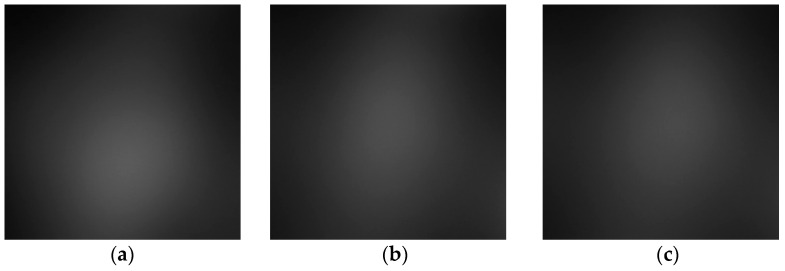
Tactile images according to elasticity: (**a**) 110 kPa, (**b**) 80 kPa, (**c**) 50 kPa.

**Figure 20:**
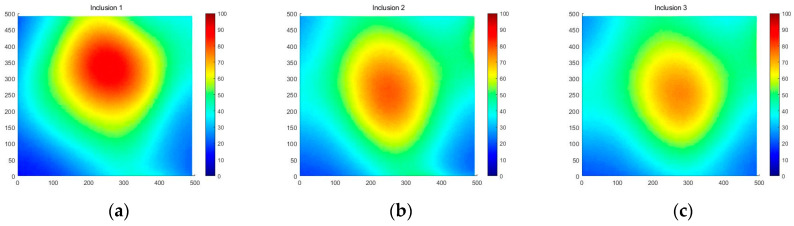
Two-dimensional colormap simulations according to elasticity: (**a**) 110 kPa, (**b**) 80 kPa, (**c**) 50 kPa.

**Figure 21:**
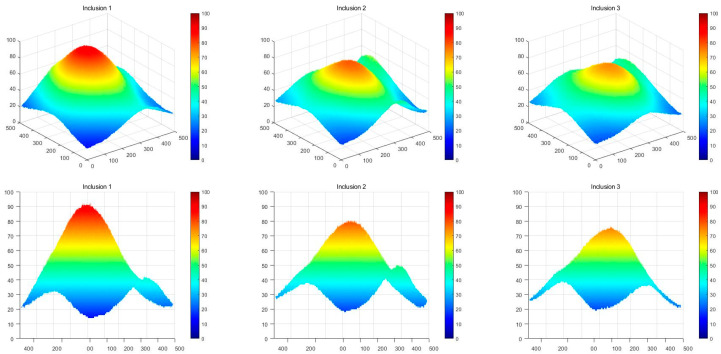
Three-dimensional elastic-image simulation according to the degree of elasticity.

**Figure 22:**
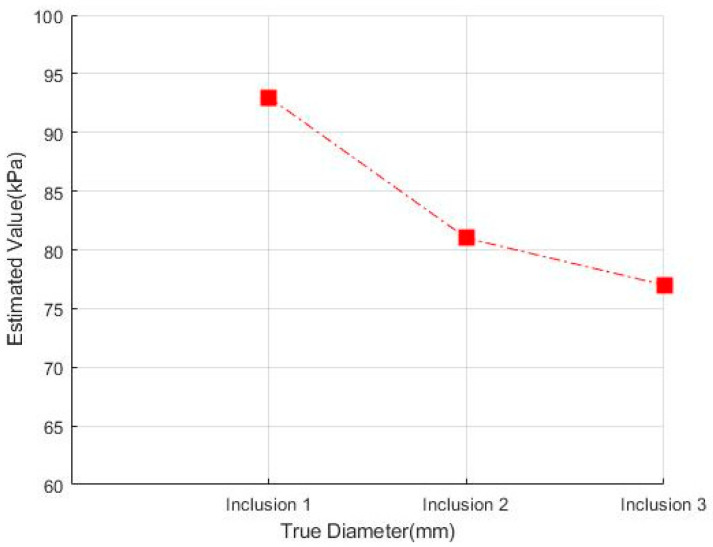
Z-score graph according to the degree of elasticity.

Figure 18 shows that the three phantoms, Inclusion 1, Inclusion 2, and Inclusion 3, all had the same nodule depth of 5 mm and size of 12 mm, and that they were fabricated with three different elasticity values: 110 kPa, 80 kPa, and 50 kPa, respectively. This measuring was using a Wavelength Dispersive X-Ray Fluorescence Spectrometer (XRF-1800, Shimadzu, Kyoto, Japan) at Intelligent Construction System Core-Support Center, Keimyung University, Republic of Korea. Normal tissues (50 kPa) and malignant tissues (110 kPa) were represented under compression by the tissue’s modulus of elasticity. According to the results of the tactile-image analysis in Figure 19, the greater the elasticity, the higher the intensity response of the gray scale. Furthermore, as a consequence of using the colormap, Figure 20 shows that the greater the stiffness, the redder the center became. Furthermore, when the 3D model was used, the highest averages of the three phantoms were 93 kPa, 81 kPa, and 77 kPa, respectively—demonstrating that the greater the stiffness, the higher the Z-axis value (Figure 21). Figure 22 shows the graph of the highest z-axis value and shows that the difference can be easily determined.

#### 4.1.2. Inclusion-Depth Phantom

**Figure 23:**
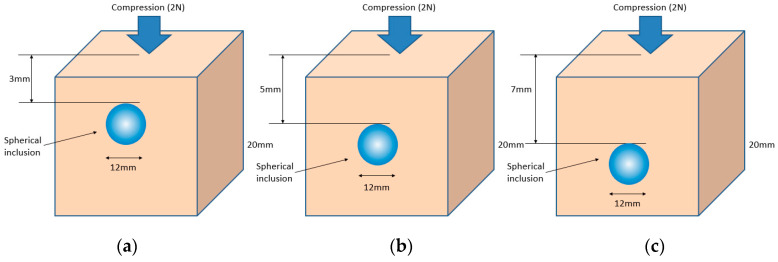
(**a**) 3-mm-deep tissue included, (**b**) 5-mm-deep tissue included, (**c**) 7-mm-deep tissue included.

**Figure 24:**
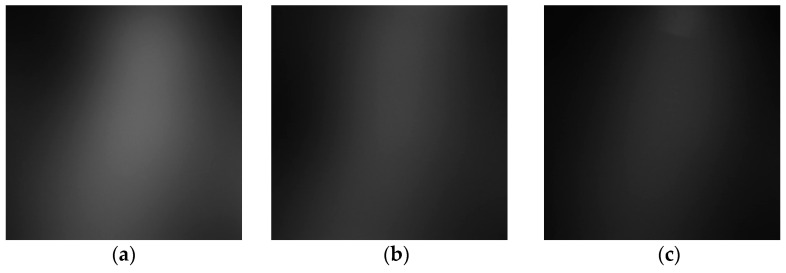
Tactile sensation images by depth: (**a**) 3 mm, (**b**) 5 mm, (**c**) 7 mm.

**Figure 25:**
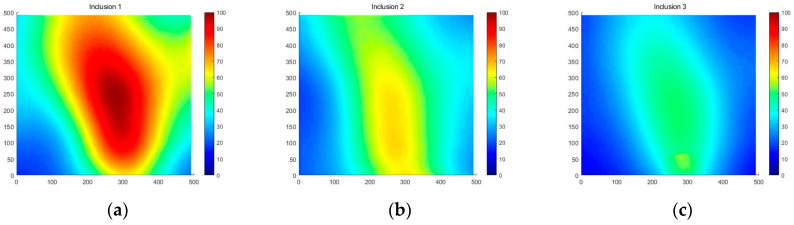
Two-dimensional colormap simulation according to depth: (**a**) 3 mm, (**b**) 5 mm, (**c**) 7 mm.

**Figure 26:**
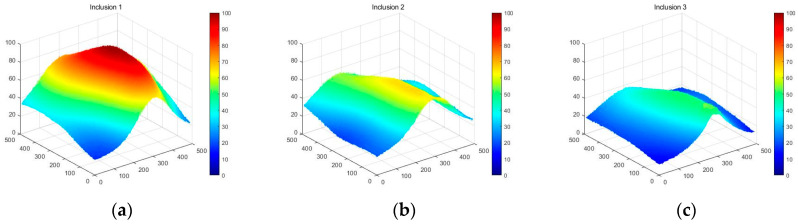
Three-dimensional elasticity-image simulation by depth: (**a**) 3 mm, (**b**) 5 mm, (**c**) 7 mm.

**Figure 27:**
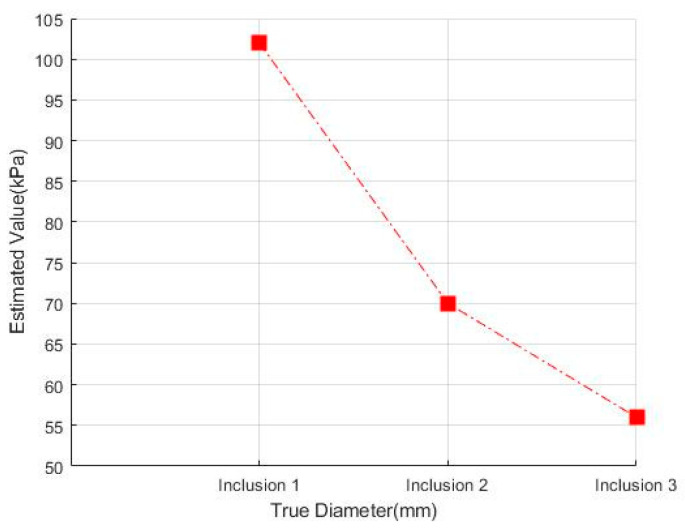
Z-score graph by depth.

Figure 23 shows that the phantoms of Inclusion 1, 2, and 3 all had the same nodule elasticity of 110 kPa and size of 12 mm—with depths of 3, 5, and 7 mm, respectively. The elasticity was also consistent with the experiment, which yielded nine tactile images. As a result of assessing the tactile images, the colormap’s sensitivity was high, since the depth was shallow and the center was drawn in the color red, as shown in Figure 24. In Figure 25, the higher the sensitivity, the higher the curvature of the 3D elastic image. The three phantoms’ highest averages were 102 kPa, 70 kPa, and 56 kPa, respectively, and the greater the depth, the lower the Z-axis value (Figure 26). Furthermore, applying the 3D model revealed that the height of the Z axis was the largest when the depth was 3 mm. Figure 27 shows the graph of the highest z-axis value and shows that the difference can be easily determined.

#### 4.1.3. Inclusion-Size Phantom

**Figure 28:**
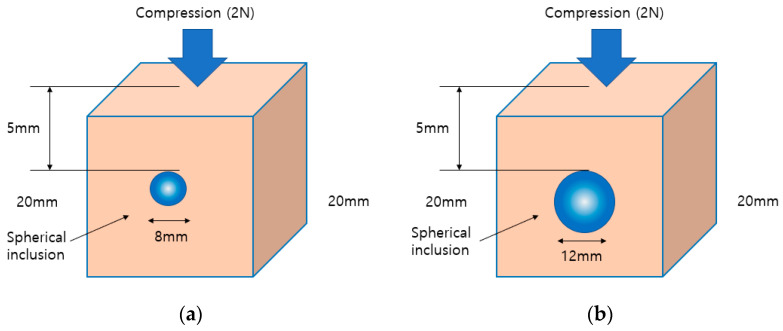
(**a**) Includes 12-mm tissues. (**b**) Includes 8-mm tissues.

**Figure 29:**
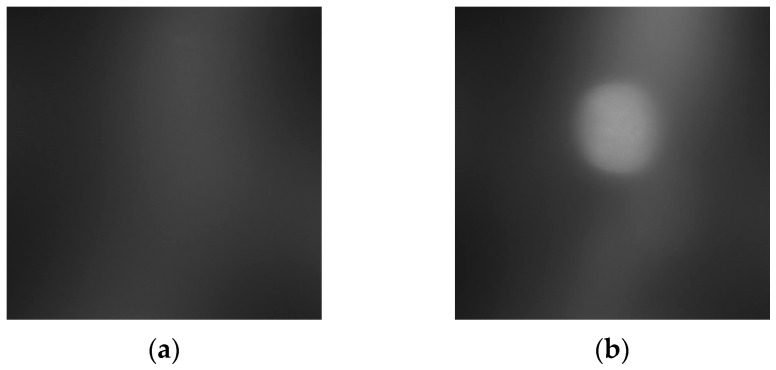
Tactile-sensation images by size: (**a**) 8 mm, (**b**) 12 mm.

**Figure 30:**
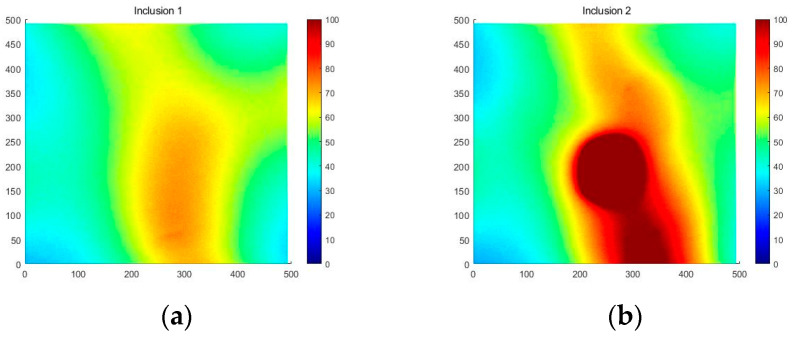
Two-dimensional colormap simulation by depth: (**a**) 8 mm, (**b**) 12 mm.

**Figure 31:**
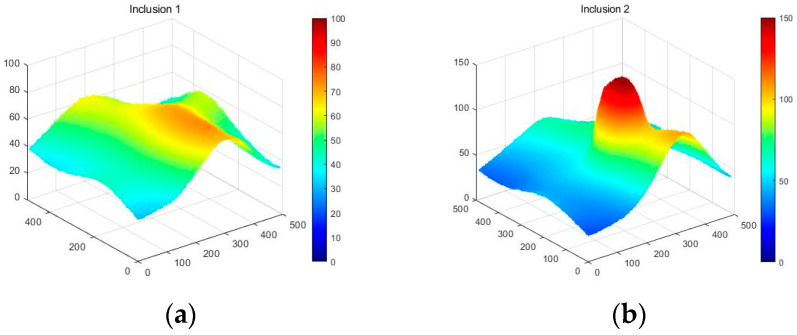
Three-dimensional elasticity-image simulation by size: (**a**) 8 mm, (**b**) 12 mm.

**Figure 32:**
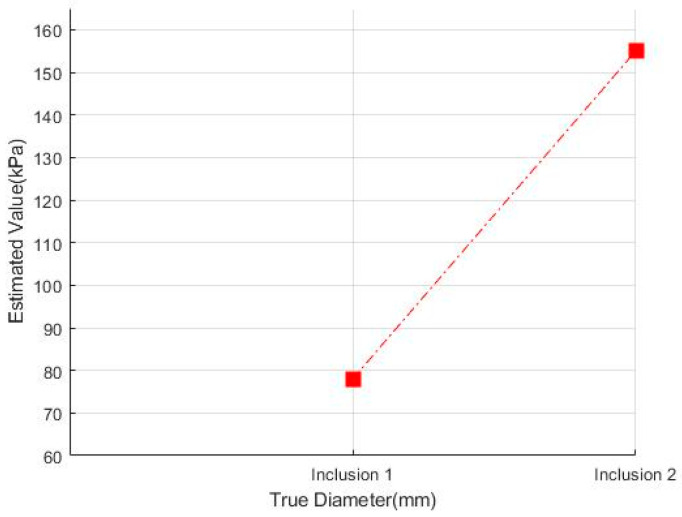
Z-score graph by size.

Figure 28 shows that the Inclusion 1 and Inclusion 2 phantoms had the same nodule elasticity of 110 kPa and depth of 5 mm but were fabricated differently, with sizes of 8 mm and 12 mm, respectively. Considering that the sizes of thyroid nodules are about 5–10 mm, they are sometimes felt by careful palpation—12 mm and 8 mm were designated as the two inclusion sizes. As a result of analyzing the tactile image in Figure 29, Figure 30 shows that the larger the size of the inclusion, the higher the sensitivity of the colormap, and the center is drawn in red. The highest averages of the two phantoms were 78 kPa and 155 kPa; it can be seen that the larger the size, the higher the Z-axis value (Figure 31). Moreover, the result of applying the 3D model indicates that the Z axis was at its highest when the size was 12 mm. Figure 32 shows the graph of the highest z-axis value and shows that the difference can be easily determined.

### 4.2. Segmentation

**Figure 33:**
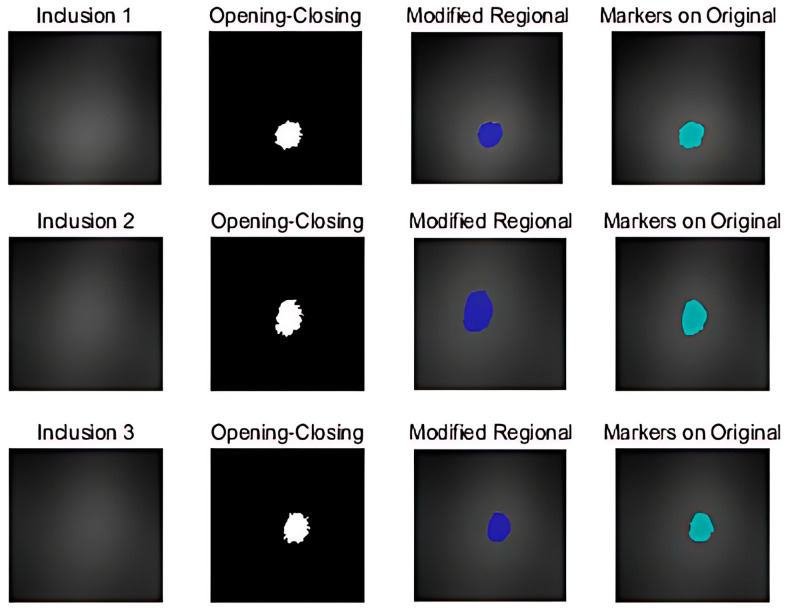
Thyroid-tumor-extraction algorithm.

By extracting nodules from thyroid ultrasound images, the proposed system instantly decreases diagnostic time and expense. Figure 33 shows an image taken by the TSIS before applying a 2D colormap and 3D elastic-image algorithms. The Tumor Extraction Algorithm was created so that medical staff could see the locations, sizes, and shapes of tumors through images. Malignant nodules have irregular margins, taller-than-wide shapes, and greater aspect ratios. This makes it easy to determine the presence of malignant tumors and to follow up on their progression or regression [13]. As reported in Section 4.1, the fuzzy-binarization approach was employed to alter the contrast of the images of the three phantoms. Furthermore, the thyroid nodule was extracted using opening and closing procedures. The last potential thyroid-nodule locations were extracted using a contour-tracing technique. The diagnostic instrument captured the image of a bell-shaped light expanding outwards. As a result, without the present preprocessing method, it was impossible to recover an accurate region, owing to over-segmentation. Therefore, this region was recreated using the morphology approach to augment the measured image. The empty space in the region, in which the presence of a thyroid nodule was suspected, was filled and the outline was drawn by connecting the adjacent areas. In addition, the objects in contact with the contour were removed to extract the ganglion site more clearly. To further clarify the potential nodule areas, unnecessary regions inside the object were eliminated. The form of the ganglion was unclear when using the conventional fuzzy-binarization approach, and there were numerous gaps in the contour, making it difficult to precisely establish the shape of the ganglion. Furthermore, the ganglion sites were accurately extracted, even for areas and sizes of skin whose texture could not be diagnosed.

### 4.3. Conclusions

**Figure 34:**
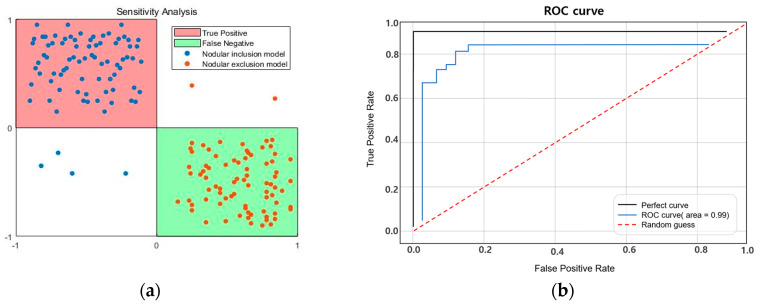
(**a**) Sensitivity analysis. (**b**) ROC curve.

We designed and tested a new TSIS capable of characterizing intratissue inclusions in this study. The thyroid tissue was exposed to pressure during scanning using the TIR principle, which caused a change in its elasticity. Using the TIR principle, the light scattering was photographed to yield high-resolution tactile images inside a multilayer optical waveguide mimicking human skin. Utilizing wave optics, the TIR was shown to be feasible and revealed clear tactile images when the multilayer silicon PDMS was crushed. As the scattered light was immediately collected by the CCD camera, the tactile resolution of the probe was determined by the camera’s resolution. The TSIS was confirmed experimentally using tissue phantoms with various inclusions for each condition. Unusual forces generated by rigid bodies were identified as bright pixels in grayscale values. The findings derived were based on the criteria of elasticity, depth, and size, and were relatively accurate in determining the presence of cancer. The highest z-score was 155 kPa when the inclusion size was 12 mm, 102 kPa when the depth was 3 mm, and 93 kPa when the elasticity was 110 kPa. The larger the inclusion size, depth, and elasticity, the greater the estimation sensitivity. As cancer advances, tissues tend to harden. Tissue stiffness can be used to differentiate between malignant and benign tumors. This is the initial stage in the development of a tactile-sensation-based diagnostic system for identifying and diagnosing thyroid cancer. As a result, the greater the stiffness, the higher the z-score; it is therefore feasible to anticipate and perform continuous follow-ups based on the relative stiffness of the intrathyroidal region. In general, malignant nodules have irregular edges, greater heights than widths, smaller sizes, and larger aspect ratios. There is a slight difference in between the morphological characteristics of hot and cold thyroid nodules. We produced and introduced an algorithm that can divide tumors in Section 4.2. By applying this algorithm from the previously photographed LED image, the tumor part can be derived. A more delicate LED auto-regulator and camera are expected to be needed to distinguish between hot and cold thyroid nodules. Through the 3D modeling of the thyroid tissue and tumor-extraction method, the 3D elasticity-imaging technique detects the tumor site and forecasts its size and elasticity. The TSIS for the thyroid in this study obtained data from 80 self-tests. We determined the true position when both the depth and elasticity and depth and size, or size and elasticity, of the nodule-containing model were correct. Furthermore, we determined the false position when both the nodule-containing model and the nodule-free model were correct. The performance test of the classifier in different contexts and conditions of elasticity, size, and depth resulted in high accuracy, with 95% sensitivity and 97% specificity, as shown in Figure 34a. Currently, the sensitivity and specificity of thyroid ultrasonography vary from reporter to reporter; they have been found to be 74–78% and 83–90%, respectively, and, according to recent reports, 93.8% and 66%, respectively [14]. On screening tests, the accuracies of neck investigations vary, depending on the technology available to the doctor and the size of the tumor. It is reported that the sensitivity of the neck to thyroid disease in asymptomatic patients is 38%, and that in about four out of six patients, thyroid nodules cannot be diagnosed [15]. When the performance assessment was expressed as a ROC curve, the performance of this TSIS was very close to the perfect curve and was drawn with high accuracy.

Consequently, this study developed a non-invasive, self-guided diagnostic device capable of extracting ganglions via quick diagnosis with minimal time and costs. This allows remote medical treatment for thyroid cancer, as well as frequent follow-up tests and recurrent monitoring. Moreover, the non-invasive computerized self-examination device does not cause radiation exposure and can be used to simply examine the thyroid without constraints on time or location. However, the developed system has a limitation in is the results it produces are derived differently from actual learned values, since no tactile-image data are obtained by probing the thyroid glands of actual patients. It seems that these limitations could be improved if actual thyroid-cancer patients were recruited and their nodules were measured and labeled, using a normal group as a control group after approval from an institutional review board. We have not yet conducted experiments on the human body, but only on the phantom. Therefore, we will proceed with animal and human experiments with approval from the IRB (Institutional Review Board). A further disadvantage is that the image taken by the TSIS cannot be checked with a portable smartphone, and must be checked with a laptop instead. Compatibility with smartphone applications would make it easy to view results using smartphones. In addition, considering the possibility of deformation during the long-term use of silicon in PDMS, we are searching for alternatives. If we create an algorithm that can predict, diagnose, and predict patient-survival rates with superior accuracy to current methods by training the system based on its application to actual human bodies, the proposed study has the potential to diagnose thyroid cancer at an early stage, with a high incidence rate. This would also have the effect of significantly reducing medical costs.

## Figures and Tables

**Table 1:** Compare to materials to manufacture PDMS.

Manufacturer	Model		Elasticity Variability	Fabricability with Elasticity Variation	Final Decision
Kafuter	k-705		Normal	X	X
Dow	SYLGARD 184	Case 1	Low	O	X
		Case 2	High	O	O

## Data Availability

The data that support the findings of this study are available from the corresponding author upon reasonable request.

## Abbreviations

**Term**	**Explanation**
PDMS	Optical-waveguide silicon
TSIS	Tactile Sensation Imaging System
Elastic Optical Waveguide	The main sensing probe of the device, composed of PDMS (high-performance silicon)
CCD Camera	Charge-coupled-device camera
2D Colormap Simulation	Algorithm for visualizing grayscale strength of tactile photograph by color
3D Elastic-Image Simulation	Algorithm for visualizing stereoscopicizing 2D colormap simulation
Z-Score Graph	Maximum height of 3D elastic-image simulation peak (z-axis)
